# Expression of *PTGS2* along with genes regulating *VEGF* signalling pathway and association with high‐risk factors in locally advanced oral squamous cell carcinoma

**DOI:** 10.1002/cam4.6986

**Published:** 2024-03-01

**Authors:** Mehta Vedant Kamal, Rama Rao Damerla, Preetiparna Parida, Mahadev Rao, Vijetha Shenoy Belle, Punit Singh Dikhit, Akhil Palod, Rinsha Gireesh, Naveena AN Kumar

**Affiliations:** ^1^ Department of Surgical Oncology, Manipal Comprehensive Cancer Care Centre, Kasturba Medical College, Manipal Manipal Academy of Higher Education Manipal Karnataka India; ^2^ Department of Medical Genetics, Kasturba Medical College, Manipal Manipal Academy of Higher Education Manipal Karnataka India; ^3^ Department of Pharmacy Practice, Centre for Translational Research, Manipal College of Pharmaceutical Sciences Manipal Academy of Higher Education Manipal Karnataka India; ^4^ Department of Biochemistry, Kasturba Medical College, Manipal Manipal Academy of Higher Education Manipal Karnataka India

**Keywords:** angiogenesis, gene expression, metastasis, Oral cancer, *PTGS2*, *VEGF*

## Abstract

**Background:**

*PTGS2* encodes cyclooxygenase‐2 (COX‐2), which catalyses the committed step in prostaglandin synthesis. Various in vivo and in vitro data suggest that COX‐2 mediates the *VEGF* signalling pathway. *In silico* analysis performed in TCGA, PanCancer Atlas for head and neck cancers, demonstrated significant expression and co‐expression of *PTGS2* and genes that regulate *VEGF* signalling. This study was designed to elucidate the expression pattern of *PTGS2* and genes regulating *VEGF* signalling in patients with locally advanced oral squamous cell carcinoma (OSCC).

**Methodology:**

Tumour and normal tissue samples were collected from patients with locally advanced OSCC. RNA was isolated from tissue samples, followed by cDNA synthesis. The cDNA was used for gene expression analysis (RT‐PCR) using target‐specific primers. The results obtained were compared with the *in silico* gene expression of the target genes in the TCGA datasets. Co‐expression analysis was performed to establish an association between *PTGS2* and *VEGF* signalling genes.

**Results:**

Tumour and normal tissue samples were collected from 24 OSCC patients. Significant upregulation of *PTGS2* expression was observed. Furthermore, *VEGFA*, *KDR*, *CXCR1* and *CXCR2* were significantly upregulated in tumour samples compared with paired normal samples, except for *VEGFB*, whose expression was not statistically significant. A similar expression pattern was observed *in silico*, except for *CXCR2* which was highly expressed in the normal samples. Co‐expression analysis showed a significant positive correlation between *PTGS2* and *VEGF* signalling genes, except for *VEGFB* which showed a negative correlation.

**Conclusion:**

*PTGS2* and *VEGF* signalling genes are upregulated in OSCC, which has a profound impact on clinical outcomes.

## Graphical abstract

This study was designed to elucidate the expression pattern of *PTGS2* and genes regulating *VEGF* signalling in patients with locally advanced oral squamous cell carcinoma. Significant upregulation of *PTGS2*, *VEGFA*, *KDR*, *CXCR1*, and *CXCR2* expression were observed in tumour samples compared with paired normal samples. This study underscores the clinical relevance of these genes, pointing to potential links between disease aggressiveness and adverse prognostic factors.

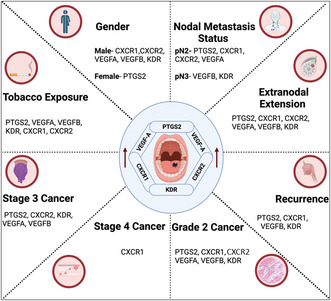

## 1 INTRODUCTION

Oral squamous cell carcinoma (OSCC) is a global health concern, with global projections reporting 202,000 cases annually.^1^ According to recent GLOBOCAN data, 354,864 new lip and oral cavity cancer cases have been diagnosed, with South and South‐Central Asia reporting the highest incidence rates.^2^ Locally advanced OSCC is treated using a multimodal approach, with standard surgery followed by adjuvant chemoradiotherapy or radiotherapy, depending on the various risk factors.^3^, ^4^ Poor disease prognosis, along with logistical issues such as a delay in access to tertiary care, often exacerbates disease progression following diagnosis and early recurrence in OSCC.^5^ Therefore, it is essential to understand the biological pathways involved in disease progression, metastasis and resistance to treatment. This will help in developing regimens that prevent metastasis and early recurrence while improving survival rates, and also help identify certain disease‐specific molecular signatures that enable us to predict treatment response.

Vascular endothelial growth factor (VEGF), a well‐known angiogenic driver molecule in both normal and pathological conditions, is widely recognised.^6^ *VEGF* signalling has a profound effect on the pathophysiology of OSCC as a crucial regulator of angiogenesis and a factor that helps differentiate new blood vessels from pre‐existing ones. Tumour cells secrete VEGF protein, which promotes angiogenesis and results in a continuous supply of oxygen and nutrients for tumour growth and proliferation. VEGF has several isoforms, including *VEGF‐A*, *VEGF‐B*, *VEGF‐C* and *VEGF‐D*, of which the most extensively investigated is *VEGF‐A*, which is largely involved in angiogenesis and vascular permeability.^7^, ^8^ Various studies have reported that *VEGFA* and *VEGFB* expression is higher in malignant tissues than in normal oral mucosa, and they play a significant role in enhancing angiogenesis and tumour growth in OSCC.^9^ *VEGFB* to a limited extent has demonstrated the ability to stimulate the development of new blood vessels.^10^, ^11^, ^12^ *VEGF* binds to specific endothelial cell receptors, VEGFR‐1 (Flt‐1) and VEGFR‐2 (KDR/Flk‐1) out of which VEGFR‐2 is thought to be the primary mediator of VEGF‐induced angiogenesis in OSCC.^13^, ^14^ By promoting the development of new blood vessels within the tumour, *VEGF* signalling increases the proliferation of cancer cells and facilitates tumour invasion into surrounding healthy tissues.^15^ *VEGF* can also stimulate lymphangiogenesis or the development of new lymphatic vessels in addition to stimulating angiogenesis. Lymphatic vessels aid in tumour metastasis by allowing cancer cells to migrate to localised lymph nodes and distant areas.^13^

Cyclooxygenase‐2 (COX‐2), also known as prostaglandin‐endoperoxide synthase 2 (*PTGS2*), has been investigated as a pro‐angiogenic because the prostaglandins generated by COX‐2 can increase the expression of angiogenic factors such as *VEGF*.^15^ In OSCC, COX‐2 overexpression can modulate angiogenesis, which stabilises the tumour microenvironment and promotes tumour progression.^16^ Poor prognosis in OSCC, including large tumours, lymph node metastases and low overall survival rates, has been linked to high COX‐2 expression.^17^ Thus, it has the potential to serve as a biomarker for determining the disease progression in patients. It also affects the tumour microenvironment by increasing vascular permeability and promoting cancer cell migration and invasion. In particular, chemokine receptors with the C‐X‐C motif (CXCR), particularly *CXCR1* and *CXCR2*, which were previously shown to be involved in inflammation and tumourigenesis, are crucial mediators of the *VEGF* signalling cascade.^18^ Immune, endothelial and cancer cells are a few examples of many cell types that contain these receptors.^19^ Owing to their role in promoting angiogenesis, *CXCR1* and *CXCR2* have been correlated with the development, invasion and metastasis of OSCC tumours.^20^ This promotes the establishment of new blood vessels, which are essential for the development and spread of tumours.

*PTGS2* and *VEGF* signalling pathway genes, including *VEGFA*, *VEGFB*, *KDR*, *CXCR1* and *CXCR2*, were found to be highly expressed in tumour samples compared to their paired normal tissues in the Cancer Genome Atlas for Head and Neck Squamous Cell Carcinoma (TCGA‐HNSCC) datasets, with *CXCR2* being the only exception, with higher expression in normal tissues than in tumour tissues.^21^ Hence, in the present study, the differential expression in tumour tissues from patients with locally advanced OSCC was examined, and the expression pattern was compared with TCGA‐HNSCC datasets computationally. It is important to note that our understanding of *VEGF* signalling in OSCC is still evolving, and our work aims to provide more insight into how the *VEGF* signalling pathway is correlated with the expression of *PTGS2*. In this study, a significant increase in *PTGS2* with *VEGFA*, *KDR*, *CXCR1* and *CXCR2* expression was observed in tumour samples compared to their paired normal samples, with the exception of *VEGFB*, whose expression was not statistically significant. Further co‐expression analysis revealed a strong positive correlation between *PTGS2* and *VEGF* signalling genes. The clinical characteristics of the cohort further assisted in a more accurate assessment of gene expression based on various risk factors.

## 2 MATERIALS AND METHODS

### 2.1 Patient enrolment and sample collection

Patients diagnosed with histopathologically proven locally advanced OSCC (pT3 and above, pN1 and above) who underwent treatment at Kasturba Hospital, Manipal from November 2021 to October 2022 with adequate renal, liver, cardiac and thyroid function, falling between the age groups of 18–80 years were included in the study. Patients with early OSCC (cT1, pT2 and cN0), patients with recurrent/second primary oral malignancy, pregnant women and patients unwilling to participate in the study were excluded. Tumour and adjacent normal tissues were collected during surgery. The tissue samples were snap‐frozen and stored at −80°C.

The demographic profile, histopathological characteristics (stage of the disease, tumour grade, nodal metastasis status, extranodal extension (ENE) status, lymphovascular invasion status, perineural invasion status etc) and data pertaining to treatment received by the patients were collected.

### 2.2 RNA isolation and cDNA synthesis

Total RNA was extracted from 50 to 100 mg of tissue using TRI Reagent (Sigma Aldrich), according to the manufacturer's protocol. The RNA purity was then assessed calorimetrically for the final preparation, considering an A260 to A280 ratio of ≥1.7, to avoid contamination. cDNA was then synthesised using the PrimeScript RT Reagent Kit (TaKaRa) and the reverse transcriptase reactions contained 500 ng of RNA samples, 2 μL of 5X PrimeScript® Buffer (for real time), 0.5 μL of PrimeScript® RT Enzyme Mix I, 4.5 μL of RNase‐free water and 1 μL of primers (0.5 μL of oligo dT primer and 0.5 μL of random 6 mers). The 10 μL reactions were incubated for 15 min at 37°C and for 5 s at 85°C and maintained at 4°C. The synthesised cDNA product was stored at −20°C for later use.

### 2.3 Differential gene expression analysis in OSCC

Real‐time polymerase chain reaction (RT‐PCR) was carried out differentially (tumour compared to paired normal) with SYBR® Premix Ex Taq II (TaKaRa) and target gene‐specific primers (*VEGFA*, *VEGFB*, *KDR*, *CXCR1*, *CXCR2* and *PTGS2*) (Table 1) using QuantStudio 5 Real‐Time PCR (Thermo Fisher). Glyceraldehyde‐3‐phosphate dehydrogenase (GAPDH) was used as the internal control. Samples were run in triplicates for 40 cycles at 95°C for 30 s and 60°C for 30 s. 2^ΔΔCT method was employed to calculate the fold quantification among paired samples. All experiments were repeated three times. Negative controls with Milli‐Q water instead of template DNA were included.

**TABLE 1:** Primers suitable for genes PTGS2, VEGFA, VEGFB, KDR, CXCR1, CXCR2 and GAPDH.

S. no.	Primer name	Primer sequence 5′ → 3′	No of bases	Product size (BP)
1	PTGS2‐F	CCTGTGCCTGATGATTGC	18	165
2	PTGS2‐R	CTGATGCGTGAAGTGCTG	18	
3	VEGFA‐F	TCTTCAAGCCATCCTGTGTG	20	102
4	VEGFA‐R	TCTGCATGGTGATGTTGGAC	20	
5	VEGFB‐F	CCTCATGATCCGGTACCCGA	20	110
6	VEGFB‐R	CCTTGGCAACGGAGGAAGCT	20	
7	KDR‐F	GGAACCTCACTATCCGCAGAGT	22	132
8	KDR‐R	CCAAGTTCGTCTTTTCCTGGGC	22	
9.	CXCR1‐F	TCCTTTTCCGCCAGGCTTACCA	22	127
10.	CXCR1‐R	GGCACGATGAAGCCAAAGGTGT	22	
11.	CXCR2‐F	TCCGTCACTGATGTCTACCTGC	22	140
12.	CXCR2‐R	TCCTTCAGGAGTGAGACCACCT	22	
13.	GAPDH‐F	CGACCACTTTGTCAAGCTCA	20	150
14.	GAPDH‐R	GAGGGTCTCTCTCTTCCTCT	20	

### 2.4 Computational gene expression and Kaplan–Meier analysis

Gene expression of *PTGS2*, *VEGFA*, *VEGFB*, *KDR*, *CXCR1* and *CXCR2*, was analysed based on patient gender, nodal metastasis status and individual cancer stages in the Cancer Genome Atlas for Head and Neck Squamous Cell Carcinoma (TCGA‐HNSCC) datasets using the University of Alabama at Birmingham Cancer Data Analysis Portal (UALCAN) (http://ualcan.path.uab.edu).^22^, ^23^ mRNA expression was expressed as transcription per million (TPM) for all selected genes. To elucidate the clinical significance of *PTGS2* and *VEGF* signalling pathway gene expression, Kaplan–Meier plots were generated computationally with log‐rank test p‐values using the GEPIA2 database (http://gepia2.cancer‐pku.cn).^24^ Stratification of patient groups was performed based on the median TPM gene expression values extracted from TCGA‐HNSCC datasets, dividing them into high and low gene expression groups.

### 2.5 Co‐expression analysis

Co‐expression analysis was conducted to ascertain the correlation between the expression patterns of *PTGS2* and genes involved in regulating *VEGF* signalling. Pearson's correlation analysis was used to assess the co‐expression relationships among the target genes.

### 2.6 Statistical analysis

All continuous variables are reported as median, interquartile range (IQR) and standard deviation (SD) after assessing the normality of the data. Wilcoxon–Mann–Whitney test was used to evaluate the expression between the tumour and normal tissue. One‐way ANOVA was performed to determine significant differences between the groups (expression based on patient sex, tobacco exposure, tumour stage and grade, ENE status, recurrence and nodal metastasis status). Pearson's correlation analysis was performed to evaluate co‐expression between target genes. Kaplan–Meier analysis was used to assess survival rates using time‐to‐event data. Patient groups with high and low expression levels were compared over a 5‐year period, and statistical significance was determined using log‐rank tests.

## 3 RESULTS

### 3.1 Patient characteristics and demographic details

Twenty‐four patients with locally advanced OSCC were enrolled in the study after obtaining informed consent. Among the enrolled patients, 16 were male and 8 were female. Two patients were reported to have a smoking habit, and 16 patients reported a habit of tobacco chewing. Of the 24 patients, 17 patients had buccal mucosa as the sub‐site of the tumour's origin, while 2 patients had gingivobuccal sulcus, 2 had retromolar trigone and 1 patient each with hard palate, tongue and upper alveolus as primary sub‐site. Eleven patients were reported to have pT4 tumours, followed by six patients with pT3 tumours and seven with pT2 tumours. Nodal metastasis was positive in 17 patients, of which 6 were pN1 followed by pN2 and pN3 in 6 and 5 patients, respectively, whereas nodal metastasis was negative (pN0) in 7 patients. Six patients were positive for ENE, seven patients were positive for lymphovascular invasion (LVI), and 15 patients were positive for perineural invasion (PNI) (details enclosed in Table 2). All recruited patients underwent surgery followed by adjuvant radiotherapy or chemoradiotherapy according to risk factors, such as advanced T‐stage(T3/T4), N‐stage (N2/N3), margin positive status, depth of invasion and PNI.

**TABLE 2:** Patient characteristics and pathological staging of the tumour (*n* = 24).

Characteristic	Value (*n* = 24)
Demographics	
Male	16
Female	8
Smoking	
Yes	2
No	22
Tobacco chewing	
Yes	16
No	8
Diet	
Vegetarian	4
Mixed	20
Alcohol	
Yes	2
No	22
Histopathology pT stage	
pT1	0
pT2	7
pT3	6
pT4	11
Histopathology pN stage	
pN0	7
pN1	6
pN2	6
pN3	5
Extranodal extension	
Positive	6
Negative	18
Perineural invasion	
Positive	15
Negative	9
Lymphovascular invasion	
Positive	7
Negative	17
Worst pathology pattern	
2	3
3	9
4	7
5	5
Tumour stage and grade (AJCC)	
Stage 3	7
Stage 4	17
Grade 1	12
Grade 2	12
Adjuvant treatment	
Chemoradiotherapy	13
Radiotherapy	9
Defaulted	2
Margin status	
Positive	1
Negative	23
Recurrence status	
Locoregional recurrence	1
Distant recurrence	5
No recurrence	18

### 3.2 Differential gene expression in locally advanced OSCC

**FIGURE 1:**
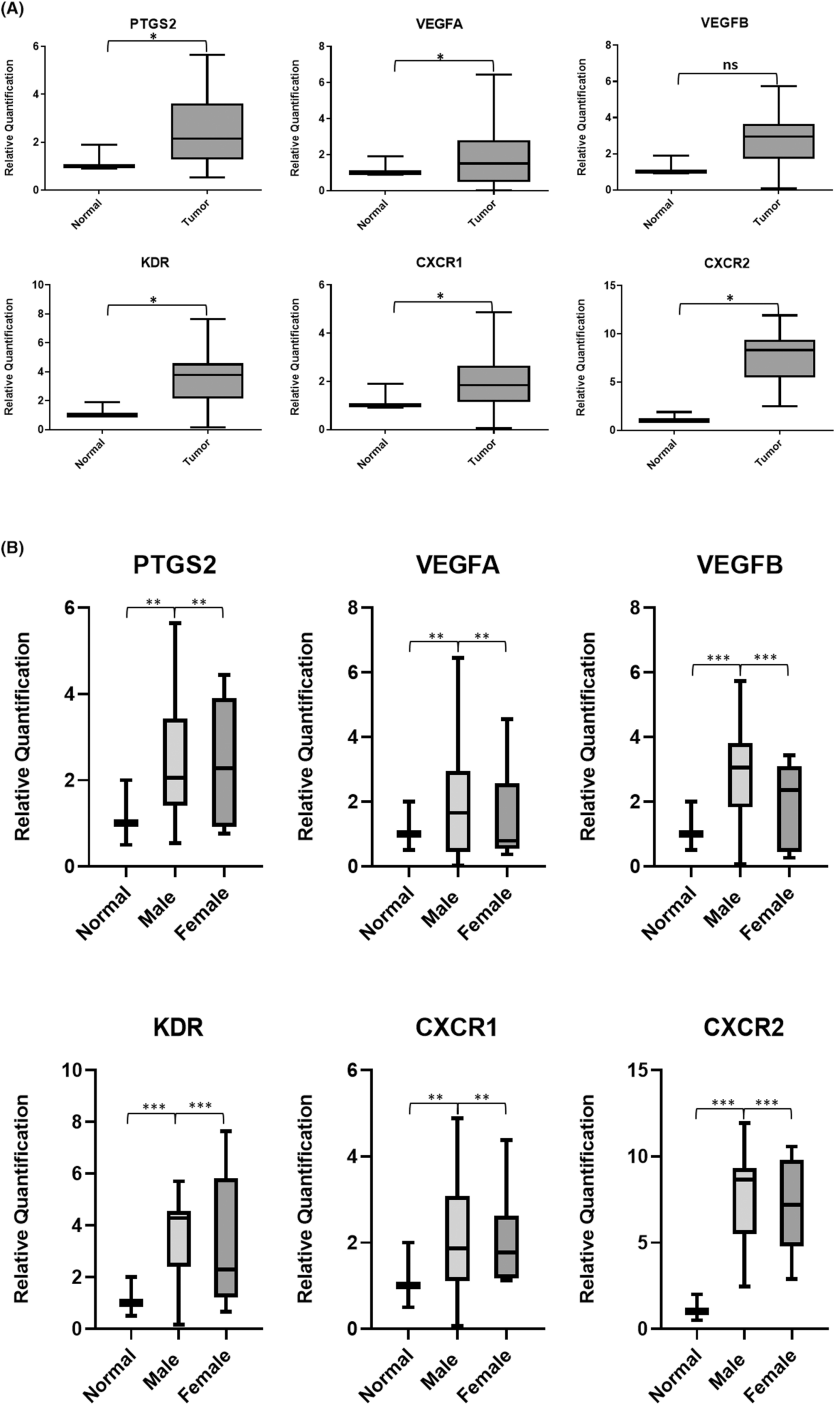
(A) The differential gene expression analysis of I. *PTGS2*, II. *VEGFA*, III. *VEGFB*, IV. *KDR*, V. *CXCR1* and VI. *CXCR2* genes in OSCC tumour samples as compared to normal. (*p < 0.05 and ^ns^ *p* >0.05) (*n* = 24). (B) The differential gene expression analysis of *PTGS2*, *VEGFA*, *VEGFB*, *KDR*, *CXCR1* and *CXCR2* genes in OSCC tumour samples (*n* = 24) based on gender stratification, that is, male (*n* = 16) and female (*n* = 8), where ***p = 0.001 and **p = 0.01.

**FIGURE 2:**
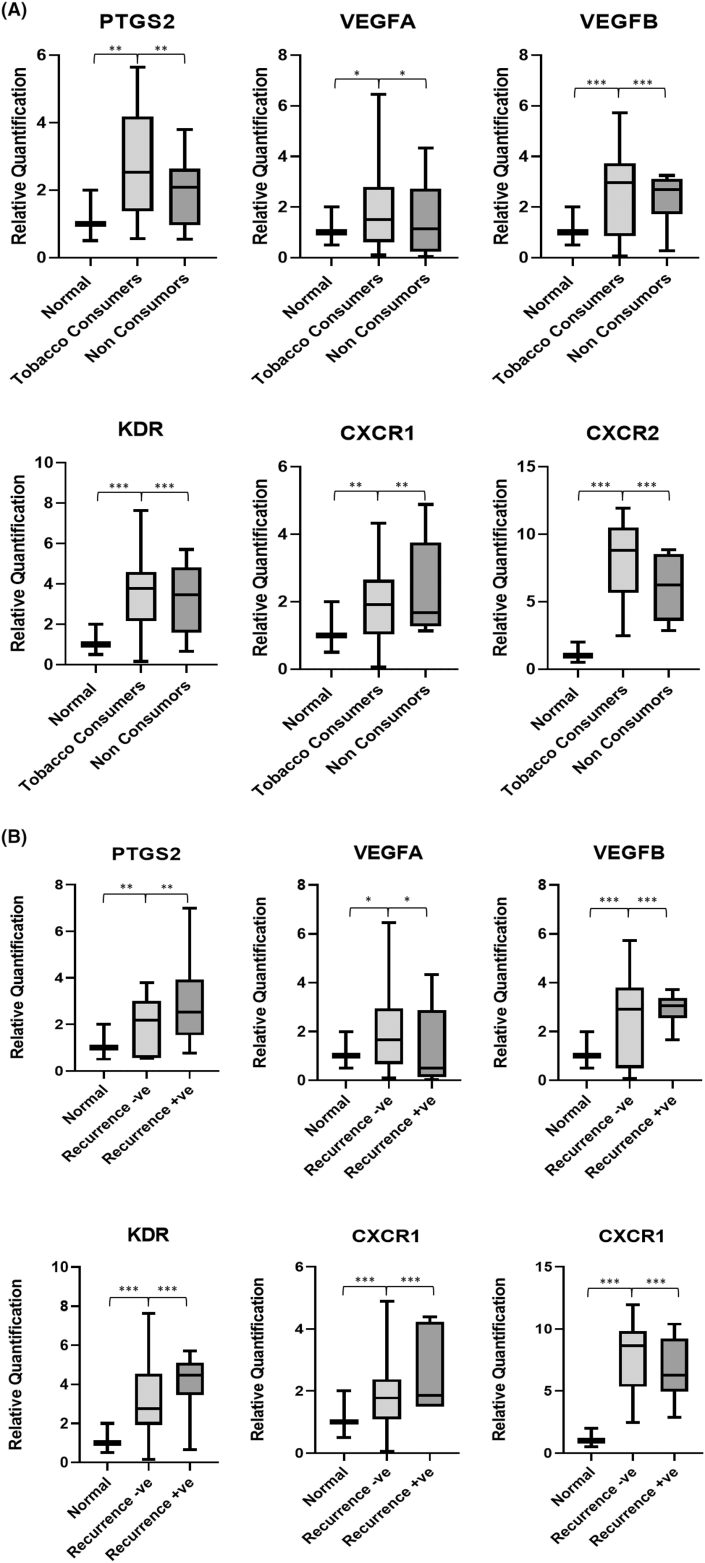
(A) The gene expression analysis of *PTGS2*, *VEGFA*, *VEGFB*, *KDR*, *CXCR1* and *CXCR2* genes in OSCC tumour samples (*n* = 24) based on smokeless tobacco consumption status, that is, tobacco consumers (*n* = 16) and non‐consumers (*n* = 8), where ***p = 0.001, **p = 0.01 and *p < 0.05. (B) The gene expression analysis of *PTGS2*, *VEGFA*, *VEGFB*, *KDR*, *CXCR1* and *CXCR2* genes in OSCC tumour samples (*n* = 24) based on recurrence status, that is, recurrence positive (*n* = 6) and recurrence negative (*n* = 18), where ***p = 0.001, **p = 0.01 and *p < 0.05.

Differential expression analysis using RT‐PCR was performed for all 24 patients. Based on the results obtained, *PTGS2*, *VEGFA*, *KDR*, *CXCR1* and *CXCR2* were found to be significantly overexpressed in tumour samples compared to paired normal samples. Similar trend was observed with *VEGFB* gene expression but the results were not statistically significant (Figure 1A). Gene expression analysis based on the patient's gender revealed that *CXCR1*, *CXCR2*, *KDR*, *VEGFB* and *VEGFA* were overexpressed in males whereas only *PTGS2* expression was observed to be expressed more in females (Figure 1B). When the cohort was stratified based on smokeless tobacco exposure, we observed that all genes, including *PTGS2*, were upregulated in patients with a smokeless tobacco chewing habit (Figure 2A). Patients with recurrence (distant and locoregional) had higher expression of *PTGS2*, *CXCR1*, *KDR* and *VEGFB* (Figure 2B).

**FIGURE 3:**
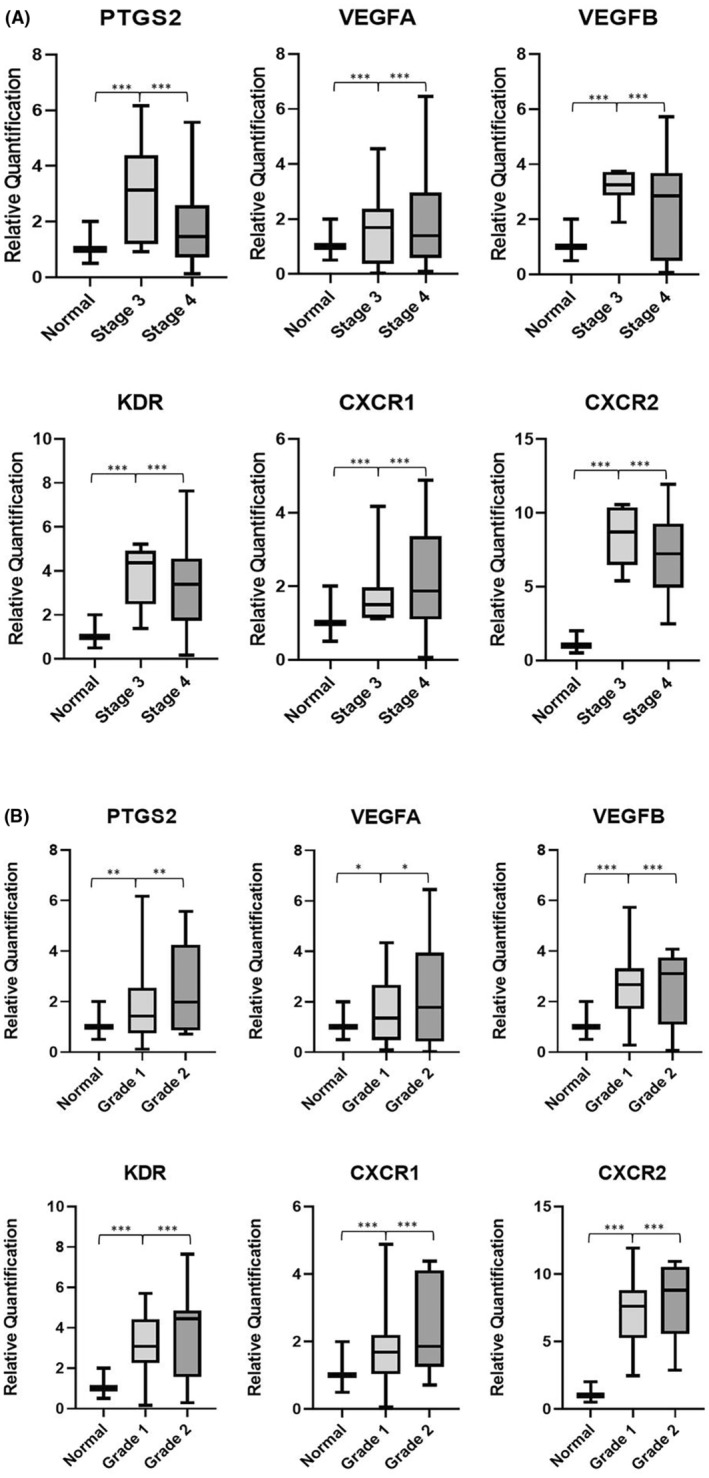
(A) The gene expression analysis of *PTGS2*, *VEGFA*, *VEGFB*, *KDR*, *CXCR1* and *CXCR2* genes in OSCC tumour samples (*n* = 24) based on cancer stage (AJCC), that is, stage 3 (*n* = 7) and stage 4 (*n* = 17), where ***p = 0.001. (B) The gene expression analysis of *PTGS2*, *VEGFA*, *VEGFB*, *KDR*, *CXCR1* and *CXCR2* genes in OSCC tumour samples (*n* = 24) based on cancer grade (AJCC), that is, grade 1 (*n* = 12) and grade 2 (*n* = 12), where ***p = 0.001, **p = 0.01 and *p < 0.05.

**FIGURE 4:**
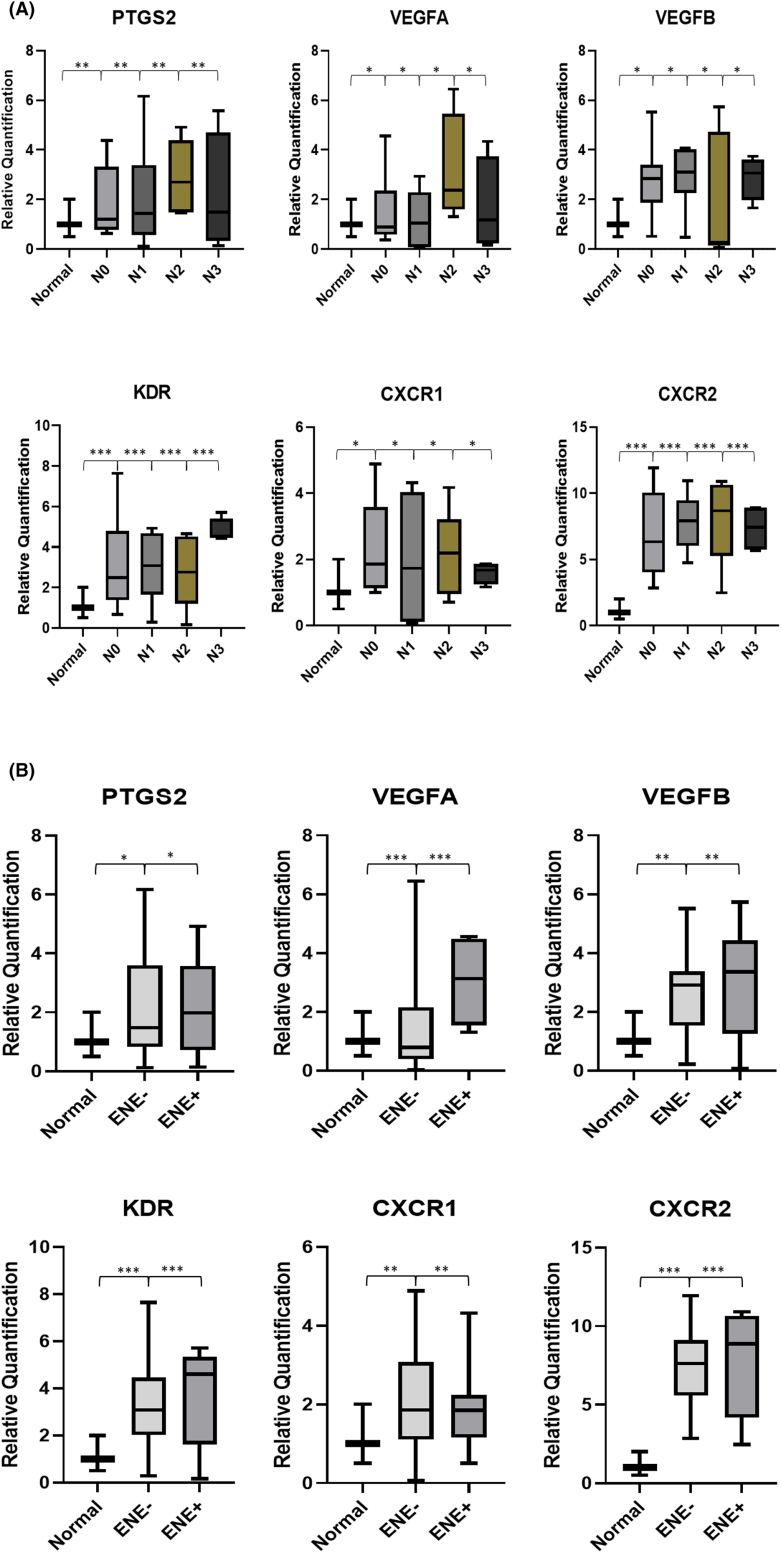
(A) The gene expression analysis of *PTGS2*, *VEGFA*, *VEGFB*, *KDR*, *CXCR1* and *CXCR2* genes in OSCC tumour samples (*n* = 24) based on nodal metastasis status, that is, pN0 (*n* = 7), pN1 (*n* = 6), pN2 (*n* = 6) and pN3 (*n* = 5), where ***p = 0.001, **p = 0.01 and *p < 0.05. (B) The gene expression analysis of *PTGS2*, *VEGFA*, *VEGFB*, *KDR*, *CXCR1* and *CXCR2* genes in OSCC tumour samples (*n* = 24) based on extranodal extension (ENE) status, that is, ENE‐positive (*n* = 6) and ENE negative (*n* = 18), where ***p = 0.001, **p = 0.01 and *p < 0.05.

Overexpression of all genes was found in stage 3 cancer patients compared to that in normal tissues, except *CXCR1* which was upregulated in patients with stage 4 disease (Figure 3A). All five genes were overexpressed in grade 2 tumours compared with those in grade 1 tumours (Figure 3B). When expression was evaluated based on the nodal metastasis status of the patients, all genes were observed to be more highly expressed in samples with positive nodal metastasis status compared to the samples with negative nodes. Furthermore, *PTGS2*, *CXCR1*, *CXCR2* and *VEGFA* were observed to be more highly expressed in patients with pN2 status, with significantly higher median expression compared to samples with pN0 status. *VEGFB* and *KDR* were observed to have a significantly higher median expression when compared to samples with pN0 in tumour samples with pN3 status (Figure 4A). When further investigating the expression based on ENE status, all genes were significantly upregulated in tumour samples with an ENE‐positive status (Figure 4B).

### 3.3 Computational gene expression and Kaplan–Meier analysis in TCGA‐HNSCC data

**FIGURE 5:**
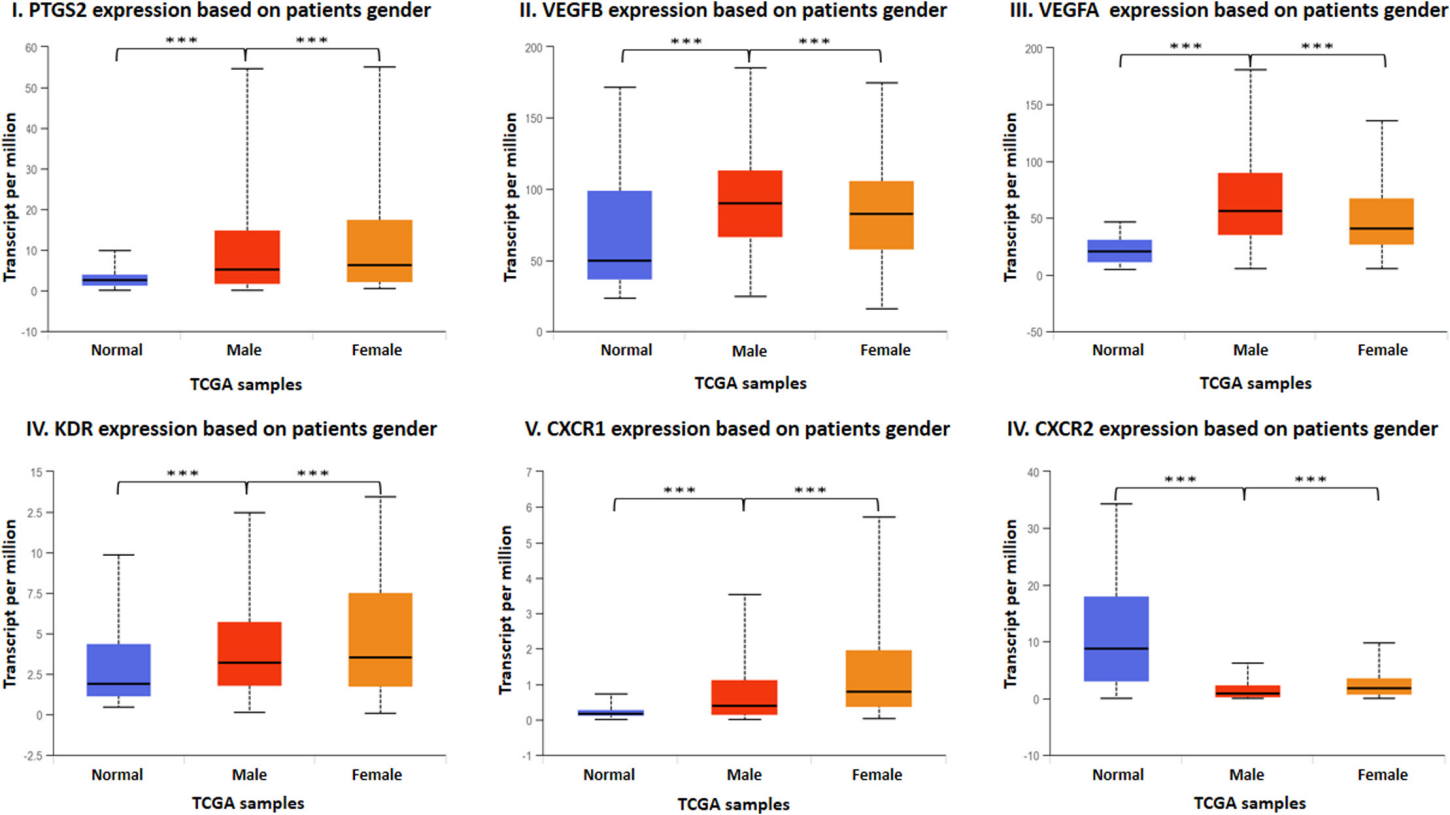
The gene expression analysis of genes, that is, I. *PTGS2*, II. *VEGFB*, III. *VEGFA*, IV. *KDR*, V. *CXCR1* and VI. *CXCR2* in head and neck squamous cell carcinoma based on patient gender as compared to normal samples using the UALCAN web server (***p = 0.001).

**FIGURE 6:**
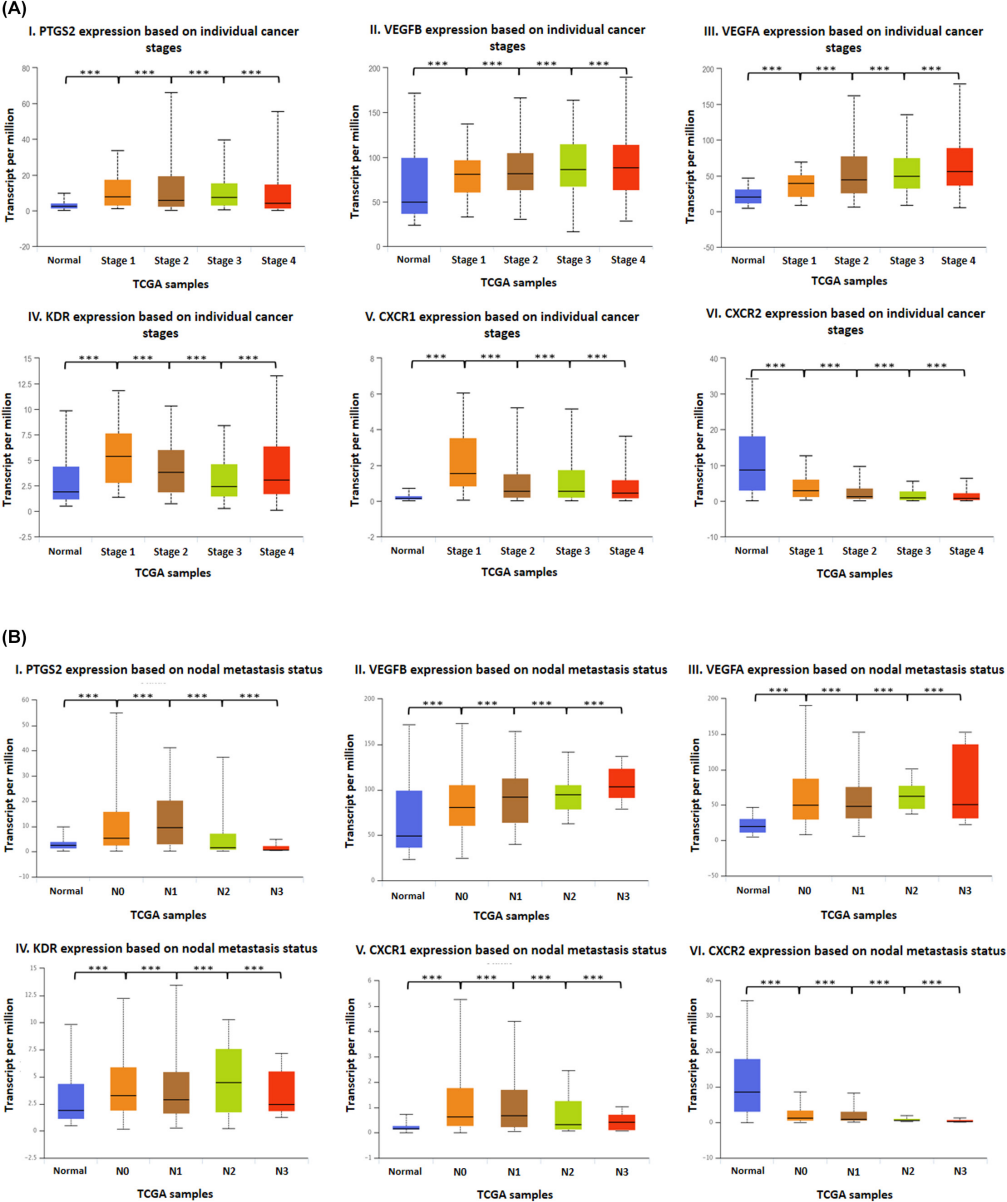
(A) The gene expression analysis of genes, that is, I. *PTGS2*, II. *VEGFB*, III. *VEGFA*, IV. *KDR*, V. *CXCR1* and VI. *CXCR2* in head and neck squamous cell carcinoma based on individual cancer stage using the UALCAN web server (***p = 0.001). (B) The gene expression analysis of genes, that is, I. *PTGS2*, II. *VEGFB*, III. *VEGFA*, IV. *KDR*, V. *CXCR1* and VI. *CXCR2* in head and neck squamous cell carcinoma is based on nodal metastasis status using the UALCAN web server (***p = 0.001).

Computational gene expression analysis based on patient gender revealed that *PTGS2*, *KDR*, *CXCR1* and *CXCR2* genes had significantly higher expression in female patients, whereas *VEGFA* and *VEGFB* genes had significantly higher expression in male patients (Figure 5). Expression based on individual cancer stages showed that *VEGFA* and *VEGFB* were highly expressed in patients with stage 4 cancer. The expression of *PTGS2* and *KDR* was significantly higher in stage 3 and stage 4 cancer, respectively. *CXCR2* genes were more highly expressed in normal samples compared to tumour samples (Figure 6A). Expression based on nodal metastasis status showed that *VEGFA* and *KDR* were significantly upregulated in samples with N2 metastasis. *CXCR1* and *PTGS2* genes had significantly higher expression in patients with N1 metastasis, whereas *VEGFB* expression was significantly higher in tumour samples with N3 metastasis. *CXCR2* expression was higher in the normal samples (Figure 6B). The computational Kaplan–Meier analysis did not achieve statistical significance. However, the analysis revealed that individuals with low expression levels of PTGS2, VEGFA, VEGFB and CXCR1 demonstrated better 5‐year survival rates than those with high expression levels. Conversely, patients expressing elevated levels of KDR and CXCR2 displayed better 5‐year survival rates compared to those expressing lower levels (see Figure S1).

### 3.4 Co‐expression analysis

**FIGURE 7:**
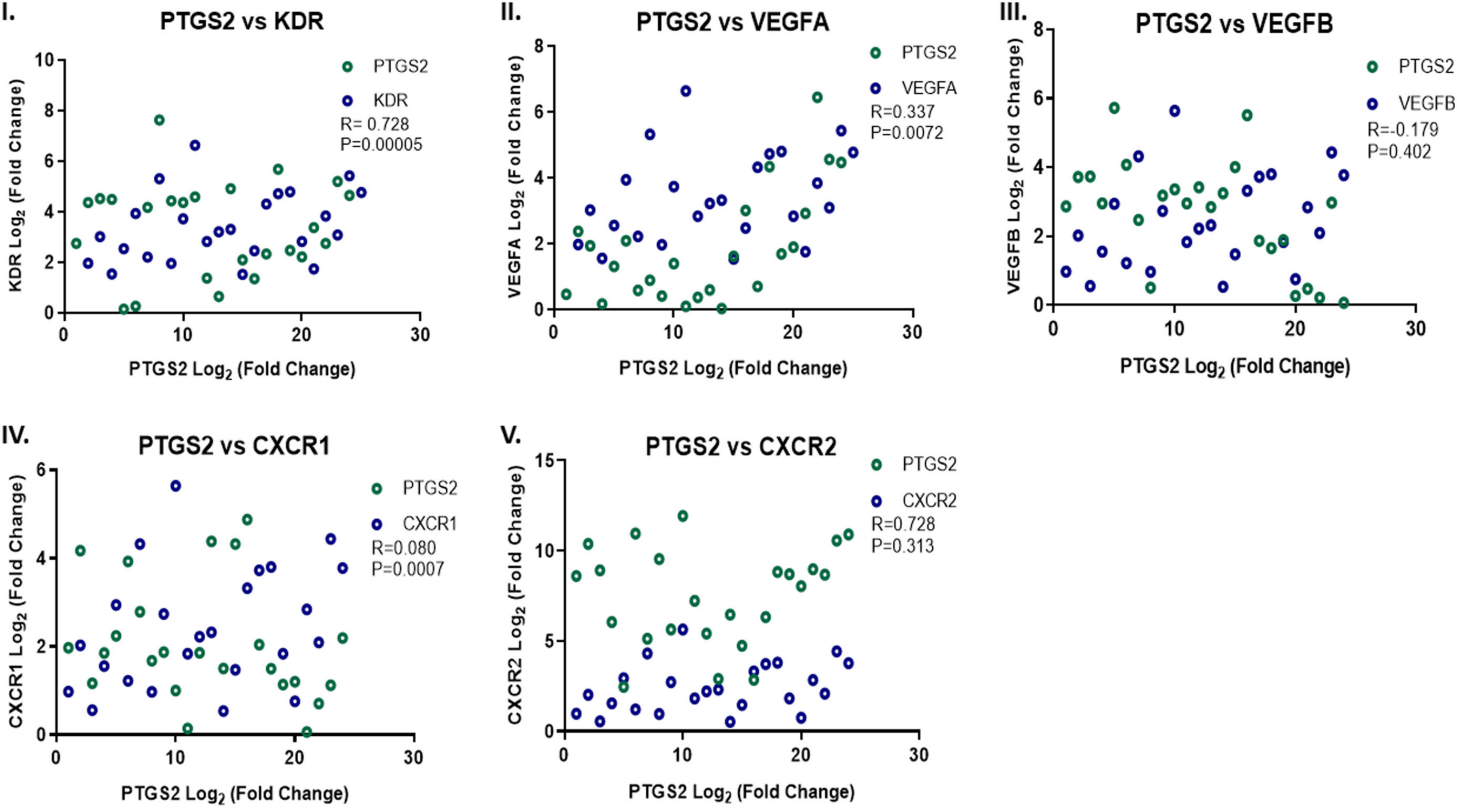
Co‐expression analysis in OSCC samples (*n* = 24) between I. *KDR* versus *PTGS2*, II. *VEGFA* versus *PTGS2*, III. *VEGFB* versus *PTGS2*, IV. *CXCR1* versus *PTGS2* and V. *CXCR2* versus *PTGS2* (*R* = Pearson correlation coefficient).

Co‐expression analysis was performed using Pearson's correlation to assess the significance of the linear association between the expression of the two genes. Co‐expression analysis was performed between *PTGS2* with *VEGFA*, *VEGFB*, *KDR*, *CXCR1* and *CXCR2*. A statistically significant positive correlation was observed between the expression of *PTGS2* with *VEGFA*, *KDR* and *CXCR1* genes. In contrast, *CXCR2* and *VEGFB* showed positive and negative correlations, respectively, but these were not statistically significant (Figure 7).

## 4 DISCUSSION

Like many other cancers, oral malignancies are significantly influenced by the *VEGF* signalling pathway. Angiogenesis is necessary for oral malignancies to establish a blood supply like other solid tumours. In a previous *in silico* study on TCGA‐HNSCC datasets (PanCancer Atlas), encompassing 523 samples, 328 displayed altered gene expression.^21^ Tumour tissues exhibited higher median expression levels, except for *CXCR2*, which showed a higher median expression in normal tissues. Analysis based on tumour grade using the UALCAN portal revealed specific expression patterns such as increased *VEGFA* expression in grade 2 and grade 3 tumours, elevated *VEGFB* expression in grade 3 tumours and high *KDR* expression in grade 4 tumours. This study further identified unique gene expression patterns associated with HPV and TP53 mutation status. HPV‐negative patients displayed significant upregulation of *PTGS2*, *KDR*, *CXCR1* and *CXCR2*, whereas *VEGFA* and *VEGFB* were more highly expressed in HPV‐positive patients. TP53‐mutated tumours exhibit elevated expression of *VEGFA*, *VEGFB*, *CXCR1* and *PTGS2*. Co‐expression analysis using cBioPortal indicated a positive correlation between *PTGS2* and *VEGF* pathway genes and a negative correlation with *VEGFB*. Gene set enrichment analysis revealed associations between various biological pathways linked to angiogenesis.^21^ However, this study has several limitations, as the identified expression patterns were confined to the populations represented in the databases, thus challenging the precision of extrapolation to broader ethnic groups. Notably, the absence of experimental validation, particularly gene expression studies on patient samples, undermines the confirmation of the results. Moreover, the study's clinical relevance was compromised by limited correlations with clinical data, underscoring the importance of a more thorough analysis in this regard. These constraints contributed significantly to further investigating the role played by *PTGS2* and *VEGF* signalling in OSCC, further offering insights into their clinical relevance, particularly in the context of locally advanced and aggressive disease.

In our study, we found that *PTGS2* and *VEGF* signalling pathway genes (*VEGFA*, *VEGFB*, *KDR*, *CXCR1* and *CXCR2*) were upregulated in OSCC tumour tissues compared to their paired normal tissues. While further investigating the expression by stratifying the patient data based on sex, pathological staging, tumour grade, ENE status and nodal metastasis status, an intriguing expression pattern was observed in all genes. All the targeted genes were more highly expressed in male patients, except for *PTGS2*, which was more highly expressed in female patients, which was also observed in TCGA‐HNSCC datasets. This can be associated with oestrogen and progesterone, which influence *PTGS2* expression, as documented in a few studies.^25^, ^26^ In the HNSCC‐TCGA data, the *KDR* and *CXCR1* genes were highly expressed in female patients. However, the available literature suggests that this might also be the case because of the female sex hormones, but it is not well established. *PTGS2* expression is upregulated in tumours following smokeless tobacco exposure, and there is ample evidence that increased *PTGS2* expression in oral tissues is associated with tobacco chewing habits.^27^ The inflammatory reaction triggered by exposure to harmful compounds in smokeless tobacco may be an underlying cause of the elevated expression of *PTGS2*.^28^ *VEGFA*, *VEGFB* and *CXCR1* expression were higher in patients with smokeless tobacco exposure, which might be in response to cellular damage caused by exposure to smokeless tobacco.^29^ *CXCR1*, which is associated with the inflammatory response brought on by exposure to carcinogens and toxins in smokeless tobacco, contributes to the greater expression of *CXCR1*.^30^ Ongoing inflammation increases the risk of cancer, whereas smokeless tobacco can cause cellular damage and localised inflammation in the oral mucosa. Increased *CXCR1* expression might be a factor in this inflammatory reaction which in the context of cancer can affect tumour growth, invasion and progression.^31^

In a stage‐wise analysis based on the American Joint Committee on Cancer (AJCC) classification (stage 3 and stage 4), all genes were upregulated in stage 3 cancers, except *CXCR1*, which showed higher expression in stage 4 tumours. Increased *CXCR1* expression in stage 4 entails disease aggressiveness, as *CXCR1* plays a crucial role in tumour cell migration and invasion.^32^ *VEGFA* and *VEGFB* genes were more highly expressed in stage 4 disease in HNSCC‐TCGA data, likely due to the highly vascularised nature of the head and neck (HN) region. With respect to the nodal metastasis status, all genes were significantly expressed in patients with positive nodal metastasis. In oral squamous cell carcinoma (OSCC), *PTGS2*‐regulated *VEGF* signalling promotes the formation of new blood and lymphatic vessels, facilitating the spread of cancer to the regional lymph nodes. Elevated *PTGS2* and *VEGF* signalling pathway expression in OSCC correlates with a higher likelihood of lymph node metastasis.^33^ *VEGFB* induces lymphangiogenesis, and *KDR* aids in maintaining lymphatic vessel structure and function.^34^, ^35^ Higher expression of all genes was observed in tumours with ENE‐positive status and grade 2 tumours, indicating a more aggressive disease.^36^, ^37^ ENE and grade 2 tumours are poor prognostic indicators associated with treatment resistance and an increased likelihood of spread or recurrence, emphasising the potential for targeted treatment strategies involving *PTGS2* and *VEGF* signalling pathway genes for optimal oncological outcomes.^38^

It is well established that *PTGS2* expression plays a significant role in inducing the *VEGF* signalling pathway, as observed in co‐expression analysis results.^39^ The increased expression of *PTGS2*, *KDR*, *VEGFB* and *CXCR1* in samples from patients with positive recurrence can be explained by their association with treatment resistance. Inflammation caused by *PTGS2* promotes the survival of cancer cells and their resistance to therapy because inflammatory cells can create inflammatory mediators that support cell survival and confer resistance to therapies that cause cell death.^40^ Overexpression of *PTGS2* can prevent apoptosis (programmed cell death), contributing to treatment resistance.^41^ Inflammatory cytokines that activate *CXCR1* can activate downstream signalling pathways that improve cancer cell survival and increase resistance to certain treatments.^42^ *CXCR1*‐associated cancer cells express invasive attributes and metastasise faster, making the cells resistant to treatment. *VEGFB* and *KDR* are often associated with angiogenesis‐mediated resistance because they stabilise the tumour microenvironment by ensuring an adequate supply of oxygen and other nutrients required for tumour growth, further adjusting the tumour microenvironment for optimum disease progression.^43^ Drugs, such as celecoxib and methotrexate (a combination used in low‐dose oral metronomic chemotherapy) and bevacizumab, a monoclonal antibody against *VEGF*, have demonstrated encouraging results in clinical trials to reduce angiogenesis and improve oncological outcomes in specific malignancies.^44^, ^45^

Our findings on the intriguing expression pattern and clinical implications of *PTGS2* and *VEGF* signalling genes provide a foundation for further investigation with comprehensive multi‐omics approach to gain deeper insights into the molecular complexity of the disease. However, the study's limitations, including the small sample size, requires validation with a larger patient cohort. Considering the potential differences in gene expression among cells, tissues and various oral cavity sub‐sites, a more comprehensive study is needed to accurately capture the overall gene expression pattern. Although useful, *in silico* predictions involve complex data processing. The unique demographic focus of this study may limit its generalisability to other ethnic groups, underscoring the need for a more extensive study with a larger and diverse patient cohort and employing a disciplinary experimental methodology for validation. As the genes analysed in this study are limited, transcriptomic and proteomic investigations (like immunohistochemistry) of genes correlated with the *PTGS2* and *VEGF* signalling pathway will further enhance our understanding of expression patterns at the translational level, providing insights into cancer biology.

## 5 CONCLUSION

This study highlights the significance of *PTGS2* and *VEGF* signalling genes in locally advanced OSCC by identifying their significant overexpression in tumour tissues. Overexpression of *PTGS2*, *VEGFA*, *VEGFB*, *KDR*, *CXCR1* and *CXCR2* was observed, with a notable association with various risk factors for OSCC. Despite the small sample size, this study emphasises the clinical relevance of these genes, suggesting potential connections to disease aggressiveness and adverse prognostic factors. Acknowledging these limitations, the findings advocate for further exploration using a multi‐omics approach to unravel molecular intricacies of OSCC. This study establishes a foundational understanding, paving the way for future research with larger and more diverse cohorts, advanced methodologies and translational insights into disease biology.

## AUTHOR CONTRIBUTIONS

**Mehta Vedant Kamal:** Conceptualization (equal); formal analysis (lead); investigation (lead); methodology (lead); validation (equal); visualization (equal); writing – original draft (lead). **Rama Rao Damerla:** Conceptualization (equal); data curation (equal); funding acquisition (supporting); investigation (supporting); methodology (supporting); project administration (supporting); resources (lead); software (lead); supervision (lead); writing – review and editing (supporting). **Preetiparna Parida:** Data curation (equal); formal analysis (supporting); investigation (equal); methodology (supporting); resources (supporting); software (supporting); validation (equal); visualization (equal); writing – original draft (supporting). **Mahadev Rao:** Conceptualization (supporting); funding acquisition (supporting); project administration (equal); resources (equal); supervision (supporting); validation (supporting); writing – review and editing (equal). **Vijetha Shenoy Belle:** Formal analysis (supporting); investigation (supporting); methodology (supporting); resources (equal); software (supporting); validation (equal); visualization (supporting); writing – review and editing (supporting). **Punit Singh Dikhit:** Data curation (equal); resources (lead); software (equal). **Akhil Palod:** Data curation (equal); resources (equal); software (equal); writing – review and editing (equal). **Rinsha Gireesh:** Data curation (equal); resources (equal); software (equal); writing – review and editing (supporting). **Naveena AN Kumar:** Conceptualization (equal); funding acquisition (lead); resources (equal); supervision (lead); writing – original draft (equal); writing – review and editing (equal).

## FUNDING INFORMATION

This work was supported by the Manipal Academy of Higher Education under faculty seed money funding [MAHE FSM No. 00000676].

## CONFLICT OF INTEREST STATEMENT

The authors declare no conflict of interest.

## ETHICS STATEMENT

The above project was reviewed by the institutional ethics committee of Kasturba Medical College and Kasturba Hospital, and approval was obtained (IEC 595/2020). Informed consent was obtained from all patients whose samples were utilised in the study.

## Supporting information


Figure S1.

## Data Availability

The computational data used in this article are freely available in The Cancer Genome Atlas (TCGA) database (https://portal.gdc.cancer.gov), UALCAN (http://ualcan.path.uab.edu) and GEPIA2 (http://gepia2.cancer‐pku.cn) database. The patient details and RT‐PCR expression raw data will be provided by the corresponding author upon request.
