# Supplementary figures and images for: Expression of PTGS2 along with genes regulating VEGF signalling pathway and association with high‐risk factors in locally advanced oral squamous cell carcinoma
Source: Cancer Med. 2024 Mar 1;13(3):e6986. doi: 10.1002/cam4.6986 (PMC10905678; doi:10.1002/cam4.6986)

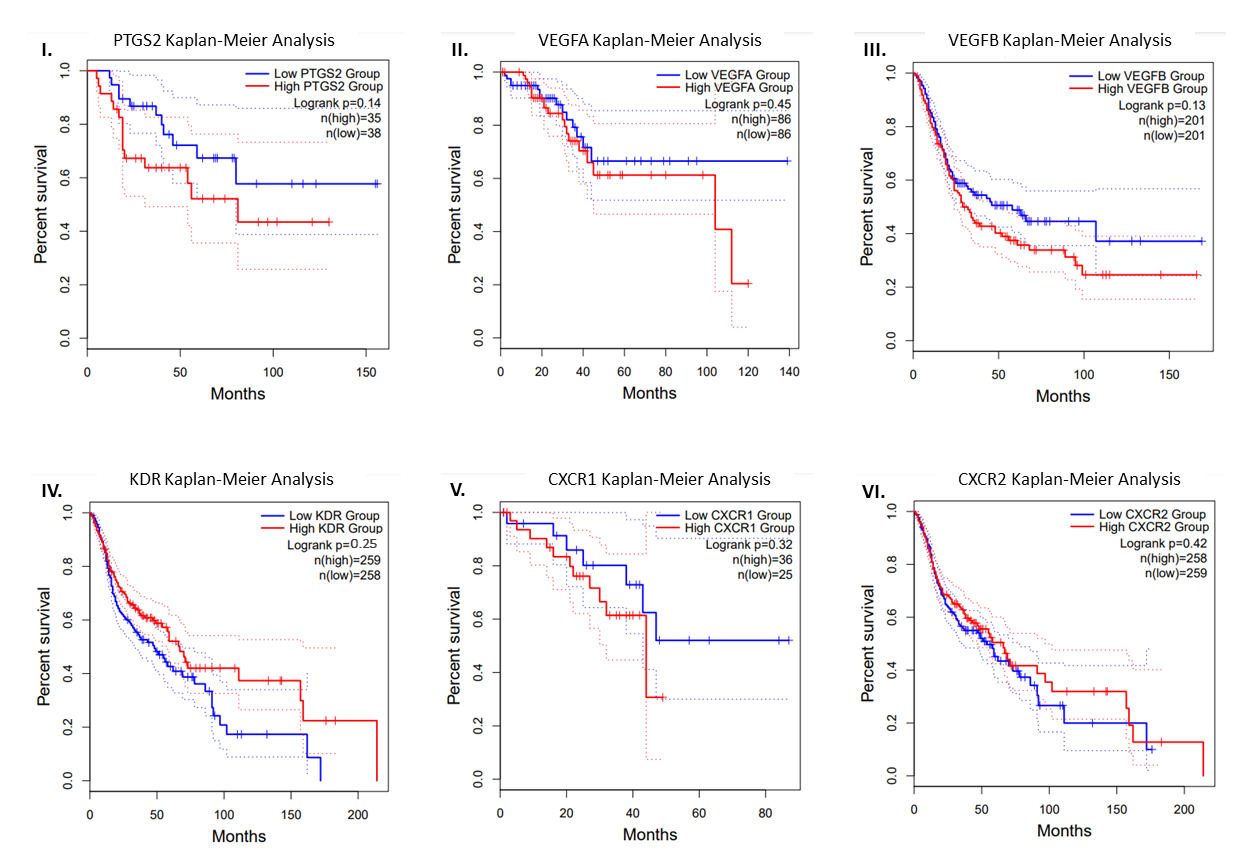

Supplement: Supplementary file 1 — Figure S1. [file CAM4-13-e6986-s001.tif]
